# Mortality-related resource utilization in the inpatient care of hypoplastic left heart syndrome

**DOI:** 10.1186/s13023-015-0355-1

**Published:** 2015-10-22

**Authors:** David A. Danford, Quentin Karels, Aparna Kulkarni, Aysha Hussain, Yunbin Xiao, Shelby Kutty

**Affiliations:** University of Nebraska Medical Center and Children’s Hospital and Medical Center, 8200 Dodge St, Omaha, NE 68114 USA; Division of Pediatric Cardiology, Albert Einstein College of Medicine, Bronx, NY USA

**Keywords:** Hypoplastic left heart syndrome, Pediatric cardiology, Congenital heart disease, Mortality-related resource utilization fraction, Outcomes

## Abstract

**Background:**

Quantifying resource utilization in the inpatient care of congenital heart diease is clinically relevant. Our purpose is to measure the investment of inpatient care resources to achieve survival in hypoplastic left heart syndrome (HLHS), and to determine how much of that investment occurs in hospitalizations that have a fatal outcome, the mortality-related resource utilization fraction (MRRUF).

**Methods:**

A collaborative administrative database, the Pediatric Health Information System (PHIS) containing data for 43 children’s hospitals, was queried by primary diagnosis for HLHS admissions of patients ≤21 years old during 2004–2013. Institution, patient age, inpatient deaths, billed charges (BC) and length of stay (LOS) were recorded.

**Results:**

In all, 11,122 HLHS admissions were identified which account for total LOS of 277,027 inpatient-days and $3,928,794,660 in BC. There were 1145 inpatient deaths (10.3 %). LOS was greater among inpatient deaths than among patients discharged alive (median 17 vs. 12, *p* < 0.0001). BC were greater among inpatient deaths than among patients discharged alive (median 4.09 × 10^5^ vs. 1.63 × 10^5^, *p* < 0.0001). 16 % of all LOS and 21 % of all BC were accrued by patients who did not survive their hospitalization. These proportions showed no significant change year-by-year. The highest volume institutions had lower mortality rates, but there was no relation between institutional volume and the MRRUF.

**Conclusions:**

These data should alert providers and consumers that current practices often result in major resource expenditure for inpatient care of HLHS that does not result in survival to hospital dismissal. They highlight the need for data-driven critical review of standard practices to identify patterns of care associated with success, and to modify approaches objectively.

## Background

Hypoplastic left heart syndrome (HLHS) remains one of the most high-risk congenital heart diseases in spite of advances in surgical techniques and perioperative management. Three decades ago the majority of babies born with HLHS died within the first days or few weeks of life, and compassionate care was the only option for this disease. In the current era, many infants with HLHS undergo a series of palliative surgeries [1]. The first of these, the Norwood Operation, usually takes place in the first week of life. In this procedure, the hypoplastic aorta is reconstructed to yield an unobstructed neo-aorta arising from the right ventricle, a shunt is placed to provide pulmonary arterial flow, and the atrial septum is resected. At approximately 4–6 months, a bidirectional superior cavopulmonary shunt (Glenn) is constructed to replace the initial shunt, which is by then outgrown. A Fontan operation, generally done at 2–4 years, incorporates inferior vena cava flow directly into the pulmonary circulation. This conventional palliation may ultimately fail, especially in older children and young adults, and cardiac transplantation may be considered at that point.

In the current era, staged surgical palliation is standard of care for HLHS in most institutions in North America (2320 Norwood operations reported by the Society of Thoracic Surgeons Congenital Heart Surgical Database in 2009) [1]. There is attrition around and between palliative surgeries, resulting in expected survival rate of 70 % at age 5 years [1, 2]. In 2011, Dean et al. documented mean hospital charges of $214,680, $82,174, and $79,549 for Norwood, Glenn, and Fontan palliations, respectively [3]. Efforts to achieve durable survival through staged palliation require sophisticated costly inpatient care [4–6]. Survivorship is improving [1, 6, 7], but the degree to which investment in this care fails to produce desired outcome is seldom studied. Some disagreements persist among physicians regarding the relative merits of encouraging parents to pursue high intensity surgical management for HLHS in the hope of a good outcome versus encouraging parents to accept a fatal neonatal outcome by providing comfort measures only [8–12].

We have developed a potentially enlightening index, the mortality-related resource utilization fraction (MRRUF), the resources expended in inpatient care which the patient does not survive divided by all inpatient care resources expended regardless of survival. The MRRUF can be conceived based on any type of measurable investment in care. True costs might be ideal, but are difficult to acquire consistently from large multi-institutional databases, so this investigation will focus on time (length of hospital stay) and money (billed charges). Our purpose is to measure the investment of inpatient care resources in the care of HLHS, and determine how much of that investment takes place during hospitalizations that have a fatal outcome.

## Methods

The Pediatric Health Information System (PHIS, Children’s Hospital Association, Overland Park, Kansas) is an administrative database containing comprehensive inpatient, observation, ambulatory surgery, and emergency department data from 43 not-for-profit children’s hospitals belonging to an alliance of free-standing pediatric hospitals. For the purposes of external benchmarking, participating hospitals provide discharge/encounter data, including demographics, International Classification of Diseases, Ninth Revision, Clinical Modification (ICD-9-CM) diagnoses, and procedures, length of stay, and discharge status (discharged to home, rehabilitation facility, nursing facility, or inpatient death). Data are deidentified at the time of data submission, and are subjected to bimonthly coding consistency reviews and quarterly data quality reports to ensure data quality, through a joint effort between the Child Health Corporation of America and participating hospitals.

The PHIS database was queried for patients ≤21 years old with the primary diagnosis of HLHS who were admitted for inpatient care during 2004–2013. There were no exclusions, and no other inclusion criteria. Institution, patient age at hospital admission, inpatient deaths, billed charges (BC) and length of stay (LOS) were recorded. Data acquisition was at the hospital admission level and not the person level. The methodology did not account for multiple occasions of admission of the same person or for transfers within or outside of PHIS. Measures of central tendency for the data were presented as mean ± standard deviation, median, and interquartile range. The mortality-related resource utilization fraction (MRRUF) was calculated based on BC and on LOS:

1 $$ \mathrm{L}\mathrm{O}\mathrm{S}\hbox{-} \mathrm{based}\ \mathrm{MRRUF} = \frac{\mathrm{LOS}\ \mathrm{f}\mathrm{o}\mathrm{r}\ \mathrm{hospitalizations}\ \mathrm{r}\mathrm{esulting}\ \mathrm{in}\ \mathrm{f}\mathrm{atality}}{\mathrm{Total}\ \mathrm{L}\mathrm{O}\mathrm{S}} $$

2 $$ \mathrm{B}\mathrm{C}\hbox{-} \mathrm{based}\ \mathrm{MRRUF}=\frac{\mathrm{BC}\ \mathrm{f}\mathrm{o}\mathrm{r}\ \mathrm{hospitalizations}\ \mathrm{r}\mathrm{esulting}\ \mathrm{in}\ \mathrm{f}\mathrm{atality}}{\mathrm{Total}\ \mathrm{B}\mathrm{C}} $$

MRRUF was calculated for the total study population, and year-by-year, and institution-by-institution in the LOS and BC domains.

Volume of admissions for HLHS (number of admissions at any participating hospital in 10 year sample/10) and mortality rates (number of deaths/number of admissions) were calculated for each institution. Due to concerns that three institutions with extremely small volume and no mortality likely were nonrepresentative of the usual practices and patterns of care for HLHS, those were excluded. The potential relations of MRRUF (LOS and BC) and of the mortality rate to institutional volume and to year of admission were assessed using least squares regression analysis and Spearman’s rank correlation for the sample including the remaining 40 institutions.

The study population was divided into 5 age groupings for comparison, age <1, 1, 2–4, 5–13, and 14–21 years. Chi square tests were applied to compare mortality rates and multiple comparisons made among the groups. To compare MRRUF (very non-normally distributed) among the 5 age groupings, the Mann–Whitney U test was applied. Levels of statistical significance were *p* < 0.05, unless corrected for circumstances of multiple comparisons, as identified in the results. Statistical analysis was done using commercially available software (Minitab 16.1, Minitab, Inc., State College, PA, USA).

## Results

**Fig. 1:**
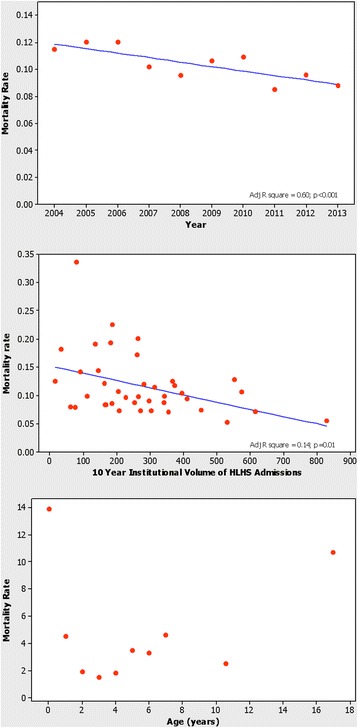
Mortality rates for Hypoplastic Left Heart Syndrome from the Pediatric Health Information System 2004–2013. Mortality rates per hospitalization for hypoplastic left heart syndrome have fallen between 2004 and 2013 (*upper panel*). The middle panel shows the significant negative correlation between institutional volume of admission for hypoplastic left heart syndrome and inpatient mortality rate. The lower panel displays the remarkable variation in inpatient mortality rate with age at admission

In total, 11,122 HLHS admissions (mean 259 ± 181 per institution) were identified which account for total LOS of 277,027 inpatient-days and $3,928,794,660 in BC. There were 1145 inpatient deaths (10.3 %). The mean, interquartile range, and median were: for patient age 1.13, 0–2, and 0 years, for LOS 24.9, 6–29, and 12 days, and for BC ($) 353,000, 86,000-392,000, and 175,000. There was a significant negative correlation between the year and the inpatient mortality rate (adjusted r^2^ = 0.060, *p* < 0.001, Fig. 1). Table 1 shows the characteristics of the dataset from which MRRUF was derived. Age at hospital admission was lower, LOS was higher, and BC were higher among non-survivors than among survivors. LOS was greater among inpatient deaths than among patients discharged alive (median 17 vs. 12, *p* < 0.0001). BC were greater among inpatient deaths than among patients discharged alive (median 4.09 × 10^5^ vs. 1.63 × 10^5^, *p* < 0.0001).

**Table 1:** Inpatient characteristics of hospitalizations with hypoplastic left heart syndrome (Pediatric Health Information System, 2004–2013)

	Number of admissions	Hospitalization duration (days)	Billed charges ($ × 10^6^)	Hospital deaths
Hospital volume (Admissions/10 years)				
< 100	398	8260	138.9	59
100–199	1441	44,777	628.9	199
200–299	2528	64,295	895.1	286
300–499	3652	95,722	1429.1	351
≥ 500	3103	63,973	836.3	250
Age at hospitalization				
< 1 year	7585	226,939	3142	1055
1 year	484	7696	126.0	22
2–4 years	2422	34,130	510.1	42
5–12 years	531	6753	121.4	18
> 12 years	100	1509	29.3	8

There was a significant negative correlation between institutional volume of admission for HLHS and inpatient mortality rate; high volume institutions had lower hospital mortality than institutions in all lower volume categories (Fig. 1). Inpatient mortality rate was remarkably higher among patients under a year old and those 15 years old or older at hospital admission than it was among those of intermediate age. There was higher mortality among the oldest and the youngest patients, and the mortality was relatively constant at lower levels at intermediate ages (Fig. 1).

**Fig. 2:**
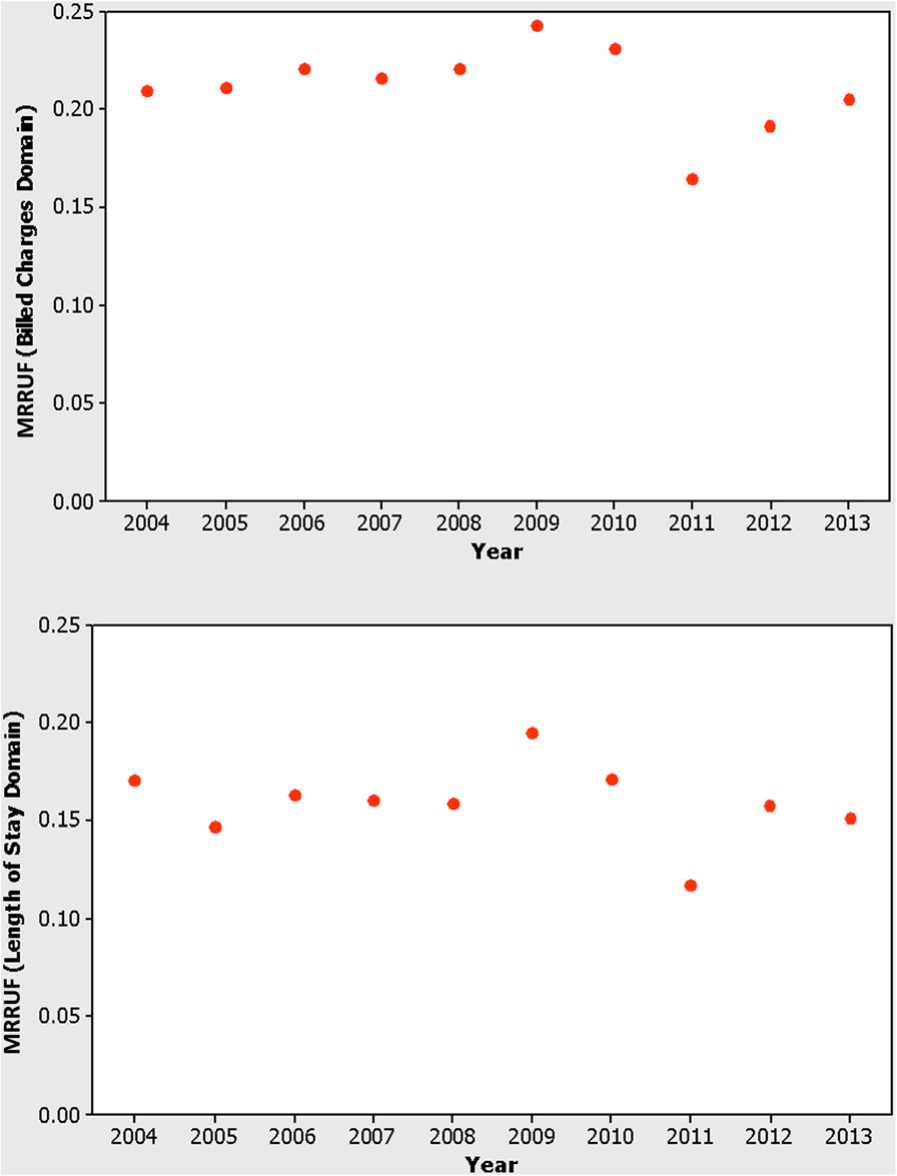
Relationship of Mortality Related Resource Utilization Fraction with Calendar Year from the Pediatric Health Information System 2004–2013. No significant association was found between calendar year and mortality related resource utilization fraction in hypoplastic left heart syndrome, either in the billed charges domain (*upper panel*) or in the length of stay domain (*lower panel*)

**Fig. 3:**
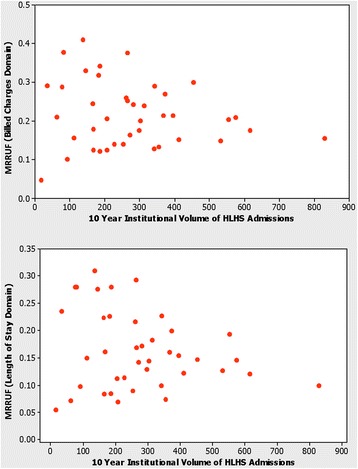
Relationship of Mortality Related Resource Utilization Fraction with Institutional Volume from the Pediatric Health Information System 2004–2013. In hypoplastic left heart syndrome, there was no significant association between institutional volume and mortality related resource utilization fraction, either in the billed charges domain (*upper panel*) or in the length of stay domain (*lower panel*)

Sixteen percent of all LOS and 21 % of all BC were accrued by patients who did not survive their hospitalization. There was no significant association between calendar year and MRRUF, either in the billed charges domain or in the length of stay domain. These MRRUF proportions year by year are shown in Fig. 2. MRRUF also did not vary significantly with institutional volume, even when potentially outlying extreme low volume institutions were excluded. Figure 3 shows the lack of significant association between institutional volume and MRRUF either in the billed charges domain or in the length of stay domain. The relation between institutional volume and MRRUF was insignificant, (Spearman’s rho = 0.039, *p* = 0.080 for billed charges, and rho = 0.097, *p* = 0.54 for length of stay).

**Fig. 4:**
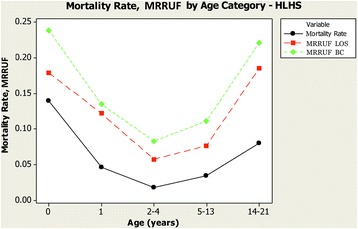
Mortality Related Resource Utilization Fraction and Mortality rate by Age Category from the the Pediatric Health Information System 2004–2013. Inpatient mortality and mortality related resource utilization fraction in hypoplastic left heart syndrome were higher among the 14–21 year olds, and those under a year old than among those of intermediate ages

MRRUF was high in the oldest patients, but those under a year old also were found to have high MRRUF relative to the intermediate age categories (Fig. 4). Comparisons of inpatient mortality rates and MRRUF are shown in Table 2.

**Table 2:** Comparisons of inpatient mortality rates and mortality-related resource utilization fraction in hypoplastic left heart syndrome (Pediatric Health Information System, 2004–2013)

Age (years)	N	Deaths (%)	Length of stay	Billed charges
<1	7585	13.9	0.178	0.238
1	484	4.5	0.121*	0.135*
2–4	2422	1.7	0.057*	0.083*
5–12	531	3.3	0.076*	0.111*
>12	100	8.0	0.185	0.220

*Indicates *p* < 0.05 relative to age < 1 year

## Discussion

As others have also documented [1, 5, 6], we observed that mortality rates in the hospital for children with HLHS have fallen in recent years, but remain substantial. The mortality is high in the very young, the age at which the Norwood operation and cavopulmonary connection are done. This is consistent with published data indicating that the Norwood operation is associated with significant risk of death [7], and that the interstage period between Norwood and cavopulmonary connection is a time of substantial attrition [13–15]. We also found that late childhood is a time of high MRRUF in HLHS, and this coincides with a time when failing Fontan physiology is well known to occur [16, 17]. Relative to these higher risk early and late periods in the modified natural history of HLHS, we observed lower mortality and MRRUF at ages during which the cavopulmonary connection and Fontan procedures occur, as well as during the early post-Fontan timeframe.

Even as mortality rates gradually decline, the costs of care for HLHS remain high [3, 4]. Our findings confirm that investment of resources in inpatient care of HLHS is high, and is disproportionately so among those who do not survive their hospitalization. Inpatient death is not the only adverse outcome known to be associated with HLHS. The findings identified here using inpatient death as a crude marker of poor outcome may substantially underestimate a larger association of more general poor outcomes in HLHS. Recent investigations have disclosed a high rate of complications from staged palliation of HLHS [16–21], including demise at home after hospital dismissal, neurologic sequelae, developmental delays, and a need in some instances for complex supportive home health care. Inpatient death, the outcome evaluated in this analysis, is therefore somewhat limited measure of the ways in which things can go wrong in HLHS care. No doubt MRRUF substantially underestimates the larger problem of investment more generally in poor outcomes, manifesting as they might not only as inpatient deaths, but also as later deaths and severe nonfatal morbidities. Nevertheless, even when the unsuccessful care is defined only by the insensitive measure of inpatient mortality, MRRUF in the care of HLHS is not small.

On a more general health policy level, the cost of care for young patients with poor prognosis from chronic conditions (cardiac and noncardiac) has attracted increasing interest in recent years [3, 22–24]. End of life care in general is known to be expensive, so it is not surprising that we find this is true for HLHS. Concerns are appropriately raised that even high levels of investment in their care may not be optimally directed toward meeting their actual needs. HLHS certainly qualifies as this kind of complex chronic condition, and the results reported here suggest at least the possibility that current patterns of investment in care may not be ideal. The ongoing search for factors associated with poor outcomes in HLHS [25, 26] may identify ways to selectively alter the approach to best serve the patients’ needs. Surgical complexity has been identified as a risk factor for high resource utilization, with an incremental increase in odds demonstrated for higher resource utilization with higher surgical risk categories [25].

Others have shown that hospital costs in patients undergoing congenital heart surgery vary significantly from center to center. These differences were most pronounced for lower complexity surgeries, and hospital surgical volume appeared to play a role [27]. It is noteworthy that MRRUF also did not vary significantly with institutional volume either in the billed charges domain or in the length of stay domain, even when potentially outlying extreme low volume institutions were excluded.

Low institutional MRRUF might or might not reflect excellence in care of HLHS. It could result from low mortality or efficient resource utilization in hopeless cases, both of which are desirable. On the other hand, it might just as easily result from institutional tendencies not to offer aggressive care for higher risk patients who might still benefit, or to give up too soon in the face of potentially fatal complications when a favorable outcome might still result from prolonged, expensive care. We therefore acknowledge that the ideal institutional MRRUF in the current era of HLHS care remains unknown, and do not argue that care for HLHS should be concentrated in centers with low institutional MRRUF.

## Limitations and future directions

The focus of this investigation was the development of the MRRUF index and its application to a specific clinical circumstance (HLHS). Two sources of potential variation in MRRUF were selected for more detailed analysis: (1) volume of HLHS admissions at the hospital providing care, and (2) patient age, as intensity of HLHS care varies somewhat predictably with age. This does not include analysis of MRRUF as a function of other potentially interesting socioeconomic markers like insurance status, race, and ethnicity. Although it stands to reason that particular surgical interventions undertaken during hospitalization would influence MRRUF via surgical mortality, it was beyond the scope of this pilot work to perform subanalysis of MRRUF based on surgical codes. This initial report is limited to HLHS, however our group is currently actively developing comparisons using PHIS datasets representing other common congenital cardiac defects. Future investigation of the role of comorbidities, including prematurity, genetic syndromes, and anomalies of other organ systems on MRRUF in HLHS and other congenital cardiac defects could provide useful data. Tracking individual patients across admissions at multiple institutions is possible so long as the institutions are PHIS participants, but for the sake of simplicity and clarity this analysis was done at the hospital admission level, not at the individual patient level.

Even as comprehensive an administrative database as PHIS lacks some clinical detail, and analyses such as this leave many interesting questions unanswered. The investigators depend on the accuracy of coding and data entry, but do not have direct quality control over either. It is well recognized that billed charges and reimbursement are not easily convertible to actual costs [28]. The MRRUF, normalized as it is to total investment, does not suffer from the inaccuracies of conversion from billed charges to actual costs, nor is it subject to distortion from inflation. However, as MRRUF is not a cost measure per se, it cannot be incorporated into a classic cost-benefit analysis. The study is limited to investments in care of HLHS, and cannot be extrapolated to the care of congenital heart disease in general. The investigators recognize that investment in inpatient fatality occurs in the care of other forms of congenital heart disease, but the results reported here carry no specific implications beyond HLHS.

## Conclusions

In conclusion, the mortality-related resource utilization fraction is very high in HLHS, and it shows no sign of falling with the passage of time, even as mortality rates gradually improve. This phenomenon is observed across institutions of all volumes of practice. Mortality-related resource utilization in HLHS is high in the very young, but peaks again to high levels in late childhood. Regardless of the large fraction of resource utilization in HLHS care that does not produce survivors, this analysis must not be construed as an argument against sophisticated invasive surgical palliative management of HLHS. Instead, these findings highlight the need for data-driven critical review of standard practices to identify patterns of care associated with success with HLHS, and to modify approaches objectively. Remarkable as the advances have been since the days when comfort measures were all that could be offered, inpatient care of HLHS carries with it substantial mortality- related resource utilization. We speculate that clinical investigations designed to discover more optimal inpatient care strategies may result in reduction in both mortality and resource utilization.

## Abbreviations

BC: Billed charges
HLHS: Hypoplastic left heart syndrome
LOS: Length of stay
PHIS: Pediatric health information system
MRRUF: Mortality-related resource utilization fraction
